# Content Validity and Psychometric Properties of the German Version of the Holm and Cordoba Urinary Tract Infection Score for Uncomplicated Urinary Tract Infections in Women: Protocol for a Validation Study

**DOI:** 10.2196/49903

**Published:** 2024-05-07

**Authors:** Katharina Piontek, Sophie Nestler, Anne Holm, John Brandt Brodersen, Christian Apfelbacher

**Affiliations:** 1 Institute of Social Medicine and Health Systems Research Medical Faculty Magdeburg Magdeburg Germany; 2 The Centre of General Practice University of Copenhagen Copenhagen Denmark; 3 The Research Unit for General Practice Region Zealand Denmark; 4 Research Unit for General Practice, Department of Community Medicine Faculty of Health Sciences UiT The Arctic University of Norway Tromsø Norway

**Keywords:** uncomplicated urinary tract infections, patient-reported outcome measures, PROM, PROMs, content validity, psychometric properties, patient reported, validity, validation, urology, urinary, urinary tract infection, UTI, psychometric, scale, score, scoring, assessment, women’s health, internal medicine, women, urinary tract infections, bacterial infection, primary care, infection

## Abstract

**Background:**

Uncomplicated urinary tract infections (UTIs) in women are among the most common bacterial infections in primary care. Given the health threats related to the overuse of antibiotics, alternative options are of increasing importance. Patient-reported outcome measures are valuable tools for including the patients’ perspective when evaluating the efficacy of these strategies. Aiming to identify a suitable instrument to measure the severity and bothersomeness of UTI symptoms in women, we performed a systematic review of the literature and identified the Holm and Cordoba Urinary Tract Infection Score (HCUTI), which measures the severity, bothersomeness, and impact of uncomplicated UTIs on daily activities. This instrument showed sufficient content validity but needs translation and further validation before it can be used in German research.

**Objective:**

For use in the German setting, we aim (1) to perform translation and linguistic validation of the HCUTI and (2) to evaluate content validity and psychometric properties of the German version of the HCUTI in a population of women with uncomplicated UTIs.

**Methods:**

The HCUTI will be translated and linguistically validated using the dual-panel method. This process involves a bilingual translation panel and a lay panel to check the comprehensibility of the translation. Content validity of the translated questionnaire will be assessed using cognitive interviews according to the criteria for good content validity as recommended by the COSMIN (Consensus-based Standards for the selection of health Measurement Instruments) group involving women with uncomplicated UTIs and health care professionals. Subsequent psychometric validation of the German version of the HCUTI in a population of women with uncomplicated UTIs will include the assessment of structural validity, internal consistency, test-retest reliability, construct validity, responsiveness, and interpretability.

**Results:**

Results of the translation and linguistic validation process and the results of the content validity study were obtained in September 2023 and will be published separately. Data on the psychometric properties of the German version of the HCUTI are anticipated in mid-2024.

**Conclusions:**

We expect that data from the content validity study will provide important suggestions for potential modifications of the HCUTI for use in the German setting. The final version of the questionnaire will be used for the assessment of its psychometric properties in a large population of women with uncomplicated UTIs.

**International Registered Report Identifier (IRRID):**

PRR1-10.2196/49903

## Introduction

### Background

Patient-reported outcome measures (PROMs) are increasingly used in medical research and patient care since researchers, clinicians, and decisions makers are becoming increasingly aware that the patients’ perspective is crucial when evaluating the efficacy of treatments and the quality of health services [1]. PROMs are standardized questionnaires for the assessment of information on health outcomes directly from the patient, including symptoms, physical, emotional, and social functioning, or multidimensional constructs such as health-related quality of life (HRQoL). Initially developed for use in research, the application of PROMs has expanded involving various domains of patient care including clinical decision-making and the evaluation of treatment strategies [1].

In primary care, uncomplicated urinary tract infections (UTIs) are among the most common bacterial infections in women and an important public health problem [2,3]. UTIs are generally self-limiting but commonly treated with antibiotics since they are bothersome, and antibiotic treatment leads to a more rapid resolution of symptoms [4,5]. Given the health threats related to conservative antibiotic treatment, the development of alternative treatment options is of increasing importance, and evaluating these strategies by considering the patients’ perspective is a high priority. For this purpose, PROMs are valuable tools. For uncomplicated UTIs, several disease-specific instruments have been developed and validated, assessing symptom burden [6,7], the impairment of daily activities [6,8], and HRQoL [6]. The selection of a PROM is guided by content-related considerations, including the construct to be measured and the target population, but it is also essential to consider the quality of the measurement properties of available instruments. Aiming to facilitate the selection of high-quality PROMs for research and patient care, the COSMIN (Consensus-based Standards for the selection of health Measurement Instruments) initiative has suggested a methodology for conducting systematic reviews of PROMs [9]. This includes the assessment of the methodological quality of single studies on measurement properties of PROMs using the COSMIN risk of bias checklist [10] and the evaluation of the quality of measurement properties of the PROMs themselves.

A systematic review of the quality of measurement properties of available PROMs for uncomplicated UTIs using the COSMIN methodology was performed [11]. Among the identified PROMs measuring symptom severity and bothersomeness, the Acute Cystitis Symptom Score (ACSS) [6] and the Urinary Tract Infection-Symptom and Impairment Questionnaire (UTI-SIQ-8) [7] can be recommended for use (COSMIN category A). Capturing the broadest spectrum of outcomes, the assessment revealed that the Holm and Cordoba Urinary Tract Infection Score (HCUTI) [12] is a promising tool that warrants further validation (COSMIN category B). The HCUTI comprises 43 items on 3 subscales measuring the following aspects over a 7-day period: symptom severity (18 items), bothersomeness (18 items), and impact on daily activities (7 items). All items are rated on a 4-point Likert scale ranging from 1=none to 4=a lot. The instrument is available in Danish and has sufficient content validity. Data on other important measurement properties, for example, structural validity, are not yet available, indicating the need for further validation studies before the instrument can be used.

### Objectives

**Figure 1:**
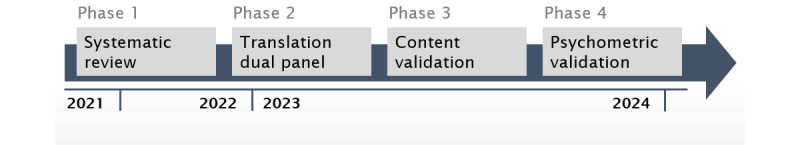
Study schedule.

The objective of this work is to perform translation and linguistic validation of the HCUTI for use in the German setting and to evaluate content validity and psychometric properties of the German version of the HCUTI in a population of women with uncomplicated UTIs. The protocol aims (1) to describe the translation process of the HCUTI from Danish into German language and the linguistic validation, (2) to describe the content validation of the translated HCUTI, and (3) to describe the psychometric validation of the German version of the HCUTI. The timeline is presented in Figure 1.

## Methods

### Translation and Linguistic Validation

The HCUTI will be translated from Danish into German language using the dual-panel method [13]. A bilingual translation panel consisting of 4 nonprofessional persons fluently speaking Danish and German will perform the translation in a web-based meeting. A bilingual person with a medical background will moderate the session under the guidance of the study team, and discussion points and decisions about the wording of the single items will be protocolized. To ensure that the original meaning of the items is maintained, the developer of the HCUTI will participate in the translation process. Based on the translations of the single members of the panel, consensus will be sought about the wording during the session. Subsequently, a lay panel involving 4 German native-speaking women who have experienced uncomplicated UTI in their lifetime will review the translation regarding comprehensibility of the instructions, items, and response options, also in a web-based meeting. Changes in wording will be documented in minutes, and the session will be additionally audio recorded for later analysis. Two members of the research team will adapt the questionnaire according to the results of the lay panel. This procedure will result in the German version of the HCUTI, for which content validity and psychometric properties will be assessed.

### Assessment of Content Validity

#### Overview

Following a qualitative approach, the content validity of the German version of the HCUTI will be assessed in cognitive interviews with affected women and interdisciplinary experts.

#### Recruitment of Participants

Women with uncomplicated UTIs will be recruited through the study team’s network. Women being at least 18 years of age who have been diagnosed with uncomplicated UTI in the past 3 years will be included. Experts from relevant disciplines including family practice, urology, gynecology, nursing, health sciences, and psychosomatic medicine will be also recruited through the study team’s network.

#### Procedure

Content validity will be assessed in accordance with the COSMIN criteria [10]. These criteria were developed in 2016 in a Delphi study among 159 experts from 21 countries including participants with expertise in qualitative research, development and evaluation of PROMs, and different professional backgrounds such as clinicians [14]. COSMIN provides a detailed, standardized, and transparent methodology, thereby promoting the selection and use of high-quality PROMs in research.

In individual semistructured interviews, women with uncomplicated UTIs (n=7) and health care professionals (n=7) will complete the questionnaire. The aim is to assess the relevance, comprehensiveness, and comprehensibility of the instructions, items, response options, and recall period from the women’s and experts’ perspectives. Two trained interviewers will conduct the interviews in a web-based meeting or in-person session following a standardized interview topic guide (Multimedia Appendix 1). The results will be documented for each item using a Microsoft Excel (Microsoft Corp) sheet. All interviews will be recorded and transcribed verbatim. Two researchers will perform data analyses using thematic analyses to identify problems related to relevance, comprehensiveness, and comprehensibility. Single items will be modified if at least 3 interviewees make a specific comment on that item. If required, the questionnaire will be adapted based on the results of the interviews, and all items will be tested in their final form in further qualitative interviews with women with uncomplicated UTIs.

### Assessment of Psychometric Properties

#### Overview

Once the content validation has been carried out and the final version of the HCUTI has been accepted by the interviewees and experts in terms of relevance, comprehensiveness, and comprehensibility, the psychometric validation study will be carried out in a quantitative web-based survey. For this purpose, the final HCUTI will be used in the target population, and the measurement properties will be analyzed using the survey data set.

#### Recruitment of Participants

**Figure 2:**
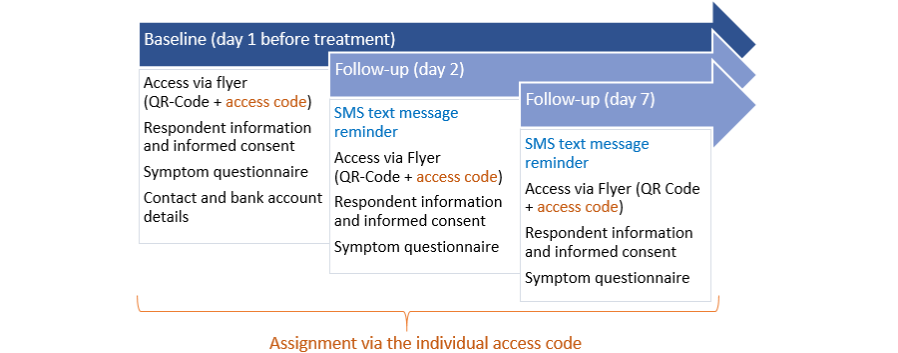
Procedure of the psychometric validation study.

A web-based survey targeting a sample size of >200 will be carried out. The study procedure is displayed in Figure 2. Women with uncomplicated UTIs will be recruited in practices for family medicine, urology, and gynecology. Women being at least 18 years of age with a confirmed diagnosis of acute cystitis or uncomplicated UTI (*International Statistical Classification of Diseases and Related Health Problems, Tenth Revision* [*ICD-10*] codes N30.0, N30.9, and N39.0) will be included. Women with complicated UTI and women with insufficient German language skills will be excluded. Participating women will complete the survey at 3 measurement points: baseline (day 1, before treatment), follow-up 1 (day 2), and follow-up 2 (day 5). Subsequent data analyses on psychometric properties will include the assessment of structural validity, internal consistency, test-retest reliability, construct validity, responsiveness, and interpretability. All analyses will be performed in accordance with the criteria of the COSMIN group [9].

### Data Analyses

#### Structural Validity

Structural validity is defined as the degree to which the scores of an instrument are an adequate reflection of the dimensionality of the construct to be measured [15]. Unidimensionality refers to whether the items in a scale or subscale measure a single construct and is an assumption for internal consistency. The HCUTI is hypothesized to measure symptom severity, bothersomeness, and impact on daily activities, suggesting the unidimensionality of the 3 subscales. Structural validity will be assessed using item response theory and Rasch analysis. Confirmatory factor analyses will be performed to test the unidimensionality of the 3 subscales. The assessed parameters according to the criteria for good measurement properties as recommended by the COSMIN group are depicted in Textbox 1.

Using Rasch analysis, the resulting dimensions will be tested for differential item functioning, which is a method for analyzing measurement equivalence. Differential item functioning of an item is present if the item response differs across groups (eg, sex and race), controlling for an estimate of the construct being measured [16].

**Parameters and criteria for the assessment of structural validity.**

- Unidimensionality: Comparative fit index or Tucker-Lewis index or comparable measure >0.95 or root mean square error of approximation <0.06 or standardized root mean square residual <0.08
- Local independency: Residual correlations among the items after controlling for the dominant factor <0.20 or Q3’s<0.37
- Monotonicity: Item characteristic curves will be generated for each item; adequate-looking graphs are required or item scalability >0.30
- Model fit: Item response theory: *χ*^2^>0.01 and Rasch: infit and outfit mean squares ≥0.5 and ≤1.5 or *Z*‐standardized values >–2 and <2

#### Internal Consistency

Internal consistency is defined as the extent to which items within an instrument measure various aspects of the same characteristic or construct and is commonly evaluated using Cronbach α [17]. Given unidimensionality, we will analyze Cronbach α for the 3 subscales of the HCUTI. Cronbach α≥.70 is considered sufficient.

#### Test-Retest Reliability

Test-retest reliability is the degree to which test scores remain unchanged when measuring a stable individual characteristic on different occasions [18]. For the assessment of test-retest reliability, patients will complete the HCUTI before treatment (baseline) and at days 2 and 5 after baseline assessment. To define stable patients, an anchor question from the ACSS [6] on change in symptom severity will be included. Patients are asked if they have perceived any changes in their symptoms. This item is rated on a 5-point Likert scale as follows: 0=now I feel back to normal (all symptoms have gone away), 1=now I feel much better (majority of symptoms has gone away), 2=now I feel only somewhat better (majority of symptoms is still present), 3=no changes, now I feel about the same (no changes in my symptoms), and 4=now I feel worse (my condition is worse).

Based on the rating, participants are categorized into 3 groups as improved, unchanged, or worsened. Test-retest reliability will be assessed for patients whose symptom severity has not changed. The degree of agreement will be assessed using intraclass correlation coefficients. An intraclass correlation coefficient >0.70 is considered sufficient.

#### Construct Validity

##### Overview

Construct validity is the degree to which a measurement instrument assesses the intended construct and involves the evaluation of the relationship between the instrument and comparator instruments measuring the same, similar, or dissimilar constructs [10]. To determine construct validity, hypotheses are formulated a priori, and these hypotheses concern comparisons with other outcome measurement instruments and differences in scores between “known” groups.

##### Comparison With Other Outcome Measurement Instruments

To determine the convergent validity of the HCUTI, the following comparator instruments will be administered:

The UTI-SIQ-8 is a self-administered questionnaire designed to assess symptom severity and bothersomeness of uncomplicated UTIs with 4 items each. The symptom severity scale is rated on a 5-point Likert scale ranging from 1=not at all to 5=very strong, and the bothersomeness scale is rated on a 5-point Likert scale ranging from 1=not at all to 5=very severe.

The ACSS comprises 4 domains assessing typical symptoms, differential diagnosis, quality of life, and additional symptoms. We will use 2 items of the quality-of-life subscale assessing the impact of UTI symptoms on daily activities and social activities. These items are rated on a 4-point Likert scale ranging from not 0=affected at all to 3=extremely affected.

The EQ-5D-5L is a generic instrument for the measurement of self-reported health status and HRQoL [19]. The EQ-5D-5L descriptive system comprises the following 5 dimensions: mobility, self-care, usual activities, pain or discomfort, and anxiety or depression. Each dimension has five response levels: 1=no problems, 2=slight problems, 3=moderate problems, 4=severe problems, and 5=unable to or extreme problems. The EQ-5D-5L further includes a vertical EQ visual analog scale capturing the respondents’ overall assessment of their health on a scale ranging from 0=worst possible health you can imagine to 100=best possible health you can imagine).

Correlations of the subscales of the HCUTI with the comparator instruments will be examined. A priori formulated hypotheses are tested using Spearman rank correlation coefficients. Following the recommendations of the COSMIN group, correlations with instruments measuring similar constructs should be ≥0.5 (high). Correlations with instruments measuring related but dissimilar constructs should be between 0.3 and 0.5 (moderate). Correlations with instruments measuring unrelated constructs should be <0.3 (low). The hypothesized associations are depicted in Table 1.

**Table 1:** Hypothesized associations between the subscales of the HCUTI^a^ and the comparator instruments.

Comparator instruments		HCUTI subscales		
		Symptom severity	Bothersomeness	Impact on daily activities
UTI-SIQ-8^b^ symptom severity		High	Moderate	Moderate
UTI-SIQ-8 bothersomeness		Moderate	High	Moderate
ACSS^c^ daily activities item		Moderate	Moderate	High
ACSS social activities item		Moderate	Moderate	High
**EQ-5D-5L**				
	Index: quality of life	Moderate	Moderate	High
	Mobility	Moderate	Moderate	Moderate
	Self-care	Moderate	Moderate	Moderate
	Usual activities	Moderate	Moderate	Moderate
	Pain or discomfort	High	Moderate	Moderate
	Anxiety or depression	Low	Low	Low
EQ VAS^d^: health status		Moderate	Moderate	Moderate

^a^HCUTI: Holm and Cordoba Urinary Tract Infection Score.

^b^UTI-SIQ-8: Urinary Tract Infection-Symptom and Impairment Questionnaire.

^c^ACSS: Acute Cystitis Symptom Score.

^d^VAS: visual analog scale.

##### Comparison Between Subgroups (Known-Groups Validity)

Since no clinical ratings or controls will be available due to the design of the study, known-groups validity of the HCUTI will be examined using Kruskal-Wallis test to compare the symptom severity score at baseline assessment between different age groups. We hypothesize no significant differences in symptom severity across age groups.

#### Responsiveness

Responsiveness to change is defined as the ability of an instrument to detect change over time in the construct to be measured [20,21]. For the assessment of responsiveness, external criteria such as patient reports are needed to determine whether the patient’s condition has improved, worsened, or not changed. For symptom severity, the anchor question from the ACSS will be used. We will further include a global rating of change item, which is rated on a 7-point Likert scale as –3=much worse, –2=moderately worse, –1=a little worse, 0=no change, +1=a little better, +2=moderately better, or +3=much better. This item will be also used to assess changes in bothersomeness and changes in impact on daily activities. Based on the rating of the anchor questions, participants are categorized into 3 groups as improved, unchanged, or worsened. Analyses on responsiveness will be performed at days 2 and 5 after the baseline assessment.

First, we will examine the sensitivity of the HCUTI to changes in patients whose severity of illness has changed. Spearman rank correlations between change scores in the anchor questions and change scores in the subscales of the HCUTI will be calculated to examine if the anchors can be considered as appropriate (>0.3) [22]. Second, receiver operating characteristic curves will be used to assess the ability of the HCUTI subscales to distinguish between patients who have experienced different changes in their condition severity. For this analysis, patients will be grouped based on the anchor question as follows: participants with improvement in symptom severity, bothersomeness, and impact on daily activities (“a little better,” “moderately better,” or “much better”) versus participants with no change or worsening in symptom severity, bothersomeness, and impact on daily activities (“no change,” “a little worse,” “moderately worse,” or “much worse”). Area under the curve values >0.70 indicate sufficient discrimination between groups [9]. The points on the curve maximizing sensitivity and specificity are considered the optimal cutoff value for differentiating between responders and nonresponders. Third, following the construct approach, we hypothesize that improvement in the symptom severity scores of the HCUTI from baseline assessment to days 2 and 5 is related to improvements in the impact on daily activities and social activities as measured with the ACSS. We further hypothesize that improvement in the symptom severity scores of the HCUTI from baseline assessment to days 2 and 5 is related to improvements in the EQ-5D-5L index score and the EuroQol visual analog scale score. Spearman correlation coefficients will be calculated to assess the degree of association between the measures of change. We expect correlations ranging from 0.30 to 0.50.

#### Interpretability

Interpretability refers to the ease of deriving meaning from an instrument’s scores. Interpretability is not considered a measurement property but is an important characteristic for the clinical application of an instrument. Data on the interpretation of changes in the scores are based on the minimal important difference (MID), which denotes the smallest score or change in score that would likely be important from the patient’s or clinician’s perspective [22]. We will estimate the MID for the 3 subscales of the HCUTI between baseline assessment and days 2 and 5 using an anchor-based approach, a distribution-based approach, and an integrated approach.

Using the anchor-based approach, the MID will be calculated by selecting patients who reported that they felt “a little worse” and “a little better” on the anchor questions. The mean change in scores of the HCUTI subscales in these groups will be calculated.

Applying the distribution-based approach, the MID is estimated based on the distribution of observed scores in the study population at baseline using 2 criteria: half SD and SE of measurement (SEM) [22]. According to these criteria, a change of more than one-half of the outcome score’s SD is considered a MID [23]. The SEM is calculated as follows: SEM=*σ*x√(1–rel), where *σ*x=SD of the scale or subscale and rel=reliability of the subscale (internal consistency). A value of 1 SEM is used as a cutoff value for determining the MID [24].

The integrated approach combines the anchor-based approach and the distribution-based approach, taking into account the advantage of both an external criterion and a measure of variability [25]. To determine the MID, the upper bound of a 1-tail 95% CI for the mean score change in the “no change” group is used. The following formula will be used: mean score change in the patient group that did not change+1.645×SE. Patients will be equally grouped as using the classical anchor-based approach.

### Ethical Considerations

The study has been approved by the Ethics Committee of the University of Magdeburg, Germany (19/23) and conforms to the principles of the Declaration of Helsinki. Written informed consent is obtained from all participants. The copyright holders gave permission to use the HCUTI for translation and validation. Data collected in the translation process; the content; and the psychometric validation study, including meeting minutes, transcripts, audio records, and survey data are stored in a pseudonymized way. An independent trusted third party at the Medical Faculty of the University of Magdeburg manages data containing personally identifiable information (consent forms and personal data required to pay incentives) and stores these data separately from the study data. A financial incentive is paid to all participants as follows: 50$ for participation in the dual panel or content validity study; 40$ for patients participating in the psychometric validation study; 100$ for physicians participating in the psychometric validation study + 10$ for each included patient.

## Results

Results of the translation and linguistic validation process and the results of the content validity study were obtained in September 2023 and will be published separately. Data on the psychometric properties of the German version of the HCUTI are anticipated in mid-2024.

## Discussion

### Expected Results

The increasing research activities in the development of alternative approaches to conventional antibiotic treatment of uncomplicated UTIs indicate the need for high-quality PROMs to evaluate the efficacy of these strategies. This study will provide data on the content validity and psychometric properties of the German version of the HCUTI. We expect that the German version of the HCUTI is a valid and reliable PROM for the assessment of the symptom severity, bothersomeness, and impact of uncomplicated UTIs on daily activities in women. A tool with sufficient measurement properties that can be used for the evaluation of novel therapeutic strategies in the German setting will be available.

### Challenges

For participation in the web-based survey for the psychometric validation of the HCUTI, strict inclusion criteria in terms of physician-diagnosed cystitis or uncomplicated UTIs according to the respective *ICD-10* codes are applied. This requires collaboration with physicians and is time-consuming. For the recruitment of participating practices, a comprehensive approach is planned, including personal contacts of the study team, personal invitation letters, contact with professional associations, conferences of local physicians, and publications in local medical journals. An incentive will be paid to all participating physicians. To keep the effort for the physicians during data collection as low as possible, they will be asked to confirm the diagnosis on a prepared form and to hand the study information out to the eligible women. The study information includes all data required for study participation, and the women can participate in the survey independently.

In view of the planned analysis, in particular regarding structural validity, responsiveness, and interpretability, a sufficient sample size must be obtained. To maximize the participation rate and minimize the dropout rate, an incentive will be paid to the participating women. For the follow-up assessment, a reminder will be sent via SMS text message to enable prompt completion of the questionnaire using a mobile device. The study procedure will be pretested to evaluate the feasibility of the procedure and the implementation of the survey in LimeSurvey (LimeSurvey GmbH).

To draw attention to our research activities, information on our study, possibilities for participation, and current results will be published on a regular basis at the Institute of Social Medicine and Health Systems Research’s website, X (formerly known as Twitter) account, and other social media platforms.

## Appendix

Multimedia Appendix 1 Interview guide.

## Abbreviations

ACSS: Acute Cystitis Symptom Score
COSMIN: Consensus-based Standards for the selection of health Measurement Instruments
HCUTI: Holm and Cordoba Urinary Tract Infection Score
HRQoL: health-related quality of life
ICD-10: International Statistical Classification of Diseases and Related Health Problems, Tenth Revision
MID: minimal important difference
PROM: patient-reported outcome measure
SEM: SE of measurement
UTI: urinary tract infection
UTI-SIQ-8: Urinary Tract Infection-Symptom and Impairment Questionnaire

## Data Availability

The data sets generated and analyzed during this study are available from the corresponding author on reasonable request.
