# Supplementary material for: Content Validity and Psychometric Properties of the German Version of the Holm and Cordoba Urinary Tract Infection Score for Uncomplicated Urinary Tract Infections in Women: Protocol for a Validation Study
Source: JMIR Res Protoc. 2024 May 7;13:e49903. doi: 10.2196/49903 (PMC11109859; doi:10.2196/49903)
Supplement: Multimedia Appendix 1 [file resprot_v13i1e49903_app1.docx]

*Welcoming*

Thank you for taking the time for the interview. With the interview, you are supporting a research project of the Institute for Social Medicine and Health Systems Research. In this project, we aim to examine the impact of acute uncomplicated urinary tract infections on daily activities in women using a symptom diary. Participating women will complete this diary on three days during their illness. Beforehand, we would like to discuss with you whether the tool is understandable, relevant and complete. We have invited you for this interview since you have experienced uncomplicated urinary tract infection in the past, and your experiences are a valuable contribution to the evaluation and potential modification of the questionnaire. The interview will probably last for about 60 minutes.

*Instruction*

Before we start, it is important for me to mention that there are no right or wrong answers. For this interview, your opinion and your experiences are crucial. I will lead the conversation. We will evaluate the questionnaire item-by-item using the criteria I have sent to you. Please open the file to you have it in front of you all the time. Alternatively, you can write the criteria down or print the file out.

| **Clarity/Comprehensibility** | |  | **Relevance** | |
| --- | --- | --- | --- | --- |
| **Code** | **Meaning** |  | **Code** | **Meaning** |
| 1 | Wording is clear |  | 1 | Activity is relevant |
| 2 | Must be presented slightly different |  | 2 | Must be presented slightly different |
| 3 | Must be presented clearly different |  | 3 | Must be presented clearly different |
| 4 | Wording is not clear |  | 4 | Wording is not clear |

For each item, we will assess the clarity and comprehensibility of the wording. Please indicate whether the item is clearly understandable for you or whether you consider minor or major changes necessary to understand the item better. Likewise, we will evaluate the relevance of each item.

We would like to record the interview for later analysis. Your data will be stored and analyzed anonymously with no linkage to your person. If you need a break during the interview, just let me know anytime. If you agree, I would start the record now.

*Assessment*

Note: In addition to the evaluation of the questionnaire according to the predefined criteria, participants are welcome to make suggestions for alternative wording or content regarding the instructions, items, response options and recall period.

General instruction:

At the beginning, I would like to ask you to read the instruction for the questionnaire aloud.

- How clear is the instruction for you?
- Are there any sentences that you consider not relevant?

Comprehensibility (item-by-item):

- Please read the sentence aloud. With regard to the response notions noted for comprehensibility and clarity, how would you rate this sentence?

Relevance (item-by-item):

- How relevant is this sentence for you? Here, too, you may use the defined response options.

Recall period und response options (assessed after the evaluation of all items):

- In retrospect, all questions refer to the last 24 hours. Do you consider this recall period appropriate?
- Let's take a closer look at the response options. Do you consider the response options appropriate and understandable?

Overall impression:

- What is your overall impression of the questionnaire?
- Are there important aspects you think that are missing in the questionnaire?
- Do you have any suggestions for improving the questionnaire?

Conclusion:

- Is there anything else you would like to add to our conversation? Perhaps an aspect that has not been mentioned so far, but you consider important?
- The interview is now finished. Thank you very much for taking the time to meet with me and I wish you all the best.
